# High DKK3 expression related to immunosuppression was associated with poor prognosis in glioblastoma: machine learning approach

**DOI:** 10.1007/s00262-022-03222-4

**Published:** 2022-05-23

**Authors:** Myung-Hoon Han, Kyueng-Whan Min, Yung-Kyun Noh, Jae Min Kim, Jin Hwan Cheong, Je Il Ryu, Yu Deok Won, Seong-Ho Koh, Jae Kyung Myung, Ji Young Park, Mi Jung Kwon

**Affiliations:** 1 Department of Neurosurgery, Hanyang University Guri Hospital, Hanyang University College of Medicine, Guri, Gyeonggi-do Republic of Korea; 2 Department of Pathology, Hanyang University Guri Hospital, Hanyang University College of Medicine, Guri, Gyeonggi-do Republic of Korea; 3 Department of Computer Science, Hanyang University, Seoul, Republic of Korea; 4 School of Computational Sciences, Korea Institute for Advanced Study, Seoul, Republic of Korea; 5 Department of Neurology, Hanyang University Guri Hospital, Hanyang University College of Medicine, Guri, Gyeonggi-do Republic of Korea; 6 Department of Pathology, Hanyang University Hospital, Hanyang University College of Medicine, Seoul, Republic of Korea; 7 Division of Cardiology, Department of Internal Medicine, Nowon Eulji Medical Center, Eulji University, Seoul, Republic of Korea; 8 Department of Pathology, Hallym University Sacred Heart Hospital, Hallym University College of Medicine, Anyang, Gyeonggi-do Republic of Korea

**Keywords:** Glioblastoma multiforme, Wnt/β-catenin signaling, Dickkopf-3, Survival, Gene

## Abstract

**Background:**

Glioblastoma multiforme (GBM) is an aggressive malignant primary brain tumor. Wnt/β-catenin is known to be related to GBM stemness. Cancer stem cells induce immunosuppressive and treatment resistance in GBM. We hypothesized that Wnt/β-catenin-related genes with immunosuppression could be related to the prognosis in patients with GBM.

**Methods:**

We obtained the clinicopathological data of 525 patients with GBM from the brain cancer gene database. The fraction of tumor-infiltrating immune cells was evaluated using in silico flow cytometry. Among gene sets of Wnt/β-catenin pathway, Dickkopf-3 (DKK3) gene related to the immunosuppressive response was found using machine learning. We performed gene set enrichment analysis (GSEA), network-based analysis, survival analysis and in vitro drug screening assays based on Dickkopf-3 (DKK3) expression.

**Results:**

In analyses of 31 genes related to Wnt/β-catenin signaling, high DKK3 expression was negatively correlated with increased antitumoral immunity, especially CD8 + and CD4 + T cells, in patients with GBM. High DKK3 expression was correlated with poor survival and disease progression in patients with GBM. In pathway-based network analysis, *DKK3* was directly linked to the *THY1* gene, a tumor suppressor gene. Through in vitro drug screening, we identified navitoclax as an agent with potent activity against GBM cell lines with high DKK3 expression.

**Conclusions:**

These results suggest that high DKK3 expression could be a therapeutic target in GBM. The results of the present study could contribute to the design of future experimental research and drug development programs for GBM.

**Graphical abstract:**

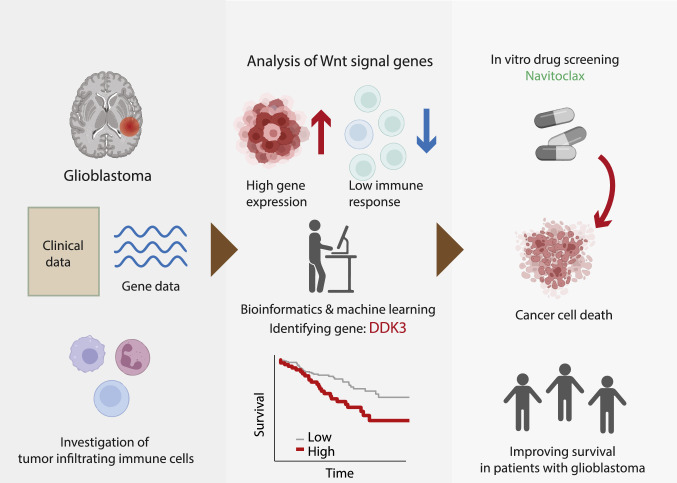

**Supplementary Information:**

The online version contains supplementary material available at 10.1007/s00262-022-03222-4.

## Introduction

The most common and lethal of all malignant central nervous system (CNS) tumors is glioblastoma multiforme (GBM) [1]. The standard treatment for glioblastoma consists of surgical resection, followed by radiation therapy and concurrent chemotherapy [2]. Despite these treatments, the median survival of glioblastoma patients is only 14.6 months [2].

A huge amount of evidence suggests that tumor cells can exhibit stem cell-like properties and that cancer stemness is a fundamentally important property of malignancy [3]. The activation of cancer stemness is associated with the limitation of antitumor immune responses [3]. Cancer stem cells can self-renew and promote tumor growth, tumor cell heterogeneity, and the induction of systemic immunosuppression [4]. GBM cancer stem cells also induce treatment resistance and an immunosuppressive GBM microenvironment [4, 5]. Wnt/β-catenin signaling is known to be associated with GBM stemness and chemoresistance [6, 7]. Therefore, we hypothesized that specific genes included in Wnt/β-catenin-associated gene sets and that are associated with immunosuppression in GBM may be related to survival and disease progression in patients with GBM.

Recently, big data analytics and next-generation sequencing have enabled analyses of genetic biomarkers, quantification of several types of tumor-infiltrating lymphoid cells and molecular pathway network-based integration of multiomics data [8–10]. The Cancer Genome Atlas (TCGA) is the world's largest publicly accessible genomic database and includes digital pathologic slides, clinicopathological information and RNA sequencing, mutation, copy number variation and methylation data [9].

**Fig. 1:**
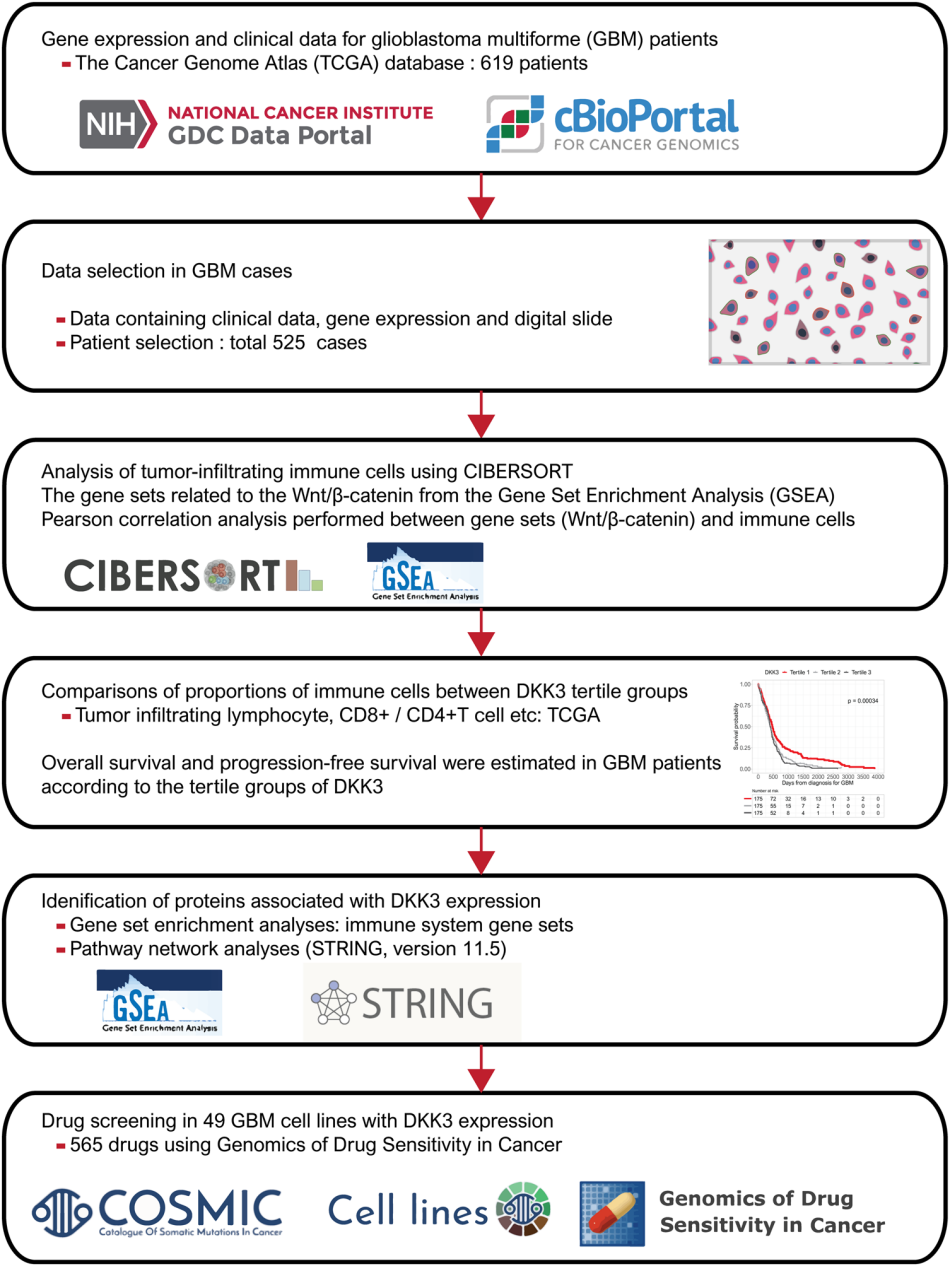
Schematic diagram depicting the plan of the study

To test the above hypothesis, we obtained the clinicopathological data of GBM patients from TCGA database. Gene sets related to the Wnt/β-catenin pathway and immune system processes were collected for gene set enrichment analysis (GSEA). CIBERSORT was used for immune cell analysis of TCGA gene expression data [11]. We also performed drug sensitivity screening in GBM cell lines using the Genomics of Drug Sensitivity in Cancer (GDSC) and the Catalog of Somatic Mutations in Cancer (COSMIC) databases. A schematic diagram depicting the analysis pipeline of the study is presented in Fig. 1.

## Methods

### Patient selection and immunohistochemistry

We obtained a total of 1,149 glioma cases of the brain (619 GBM cases and 530 low-grade glioma [LGG] cases) with known mRNA expression data from TCGA database (https://gdc.cancer.gov/about-data/publications/pancanatlas and https://www.cbioportal.org/) [12]. Normal samples as well as tumor samples with missing data were excluded from analysis. The analysis was finally performed on 525 cases with both virtual histopathological slides and clinical data (from a total of 619 GBM samples). We present the raw data of our study in Supplementary Data 1.

Immunohistochemical staining was performed to evaluate the presence or absence of tumor-infiltrating lymphocytes (TILs) in GBM human tissue diagnosed at Hanyang University Guri Hospital. Haematoxylin and eosin (H and E)-stained slides were reviewed by at least two pathologists for each case (Min and Kim). In non-necrotic tissue with TILs, immunostaining for anti-CD3 (clone LN10 Leica Biosystems, Newcastle, UK), anti-CD8 (clone 4B11 Leica Biosystems, Newcastle, UK) and anti-CD4 (clone 4B12 Leica Biosystems was performed using the Dako Autostainer Universal Staining System (DakoCytomation, Carpinteria, CA, USA) and the ChemMate™ Dako EnVision™ Detection Kit.

### Gene set enrichment analysis

GSEA is a method of analyzing and interpreting microarray and other such data based on biological information. These biological sets can be published information about a biochemical pathway or coexpression obtained in a previous experiment.

To detect significant gene sets, GSEA (version 4.1.0) was performed with 32,284 gene sets in the Molecular Signatures Database (MSigDB version 7.4) from the Broad Institute at MIT [10]. Gene sets related to the Wnt/β-catenin pathway were investigated via GSEA (https://www.gsea-msigdb.org/) (standard name, ST_WNT_BETA_CATENIN_PATHWAY; systematic name, M17761). We extracted mRNA expression data for 31 genes related to the Wnt/β-catenin pathway from the molecular signatures database. Three genes (*AXIN2, NKD2, and RPSA*) related to Wnt/β-catenin were not available in the TCGA database of GBM (Supplementary Data 1). Immunologic signature gene sets (5,219 gene sets) were tested to determine which genes were associated with *Dickkopf-3 (DKK3)*. DKK3 was entered into the Gene Identifier field in the Investigate Gene Set category of the GSEA. After clicking C7: immunologic signature gene sets and executing "compute overlaps,” we obtained immunologic signature gene sets related to DKK3. For this analysis, 1,000 permutations were utilized to calculate *p*-values, and the permutation type was set to phenotype. Significant gene sets were those with the following characteristics: false discovery rate (FDR) < 0.25 and *p* < 0.001.

### In silico cytometry and pathway-based network analysis

We analyzed tumor-infiltrating lymphocytes using deep learning-based lymphocyte classification with convolutional neural networks in whole-slide image analysis and identified immune subtypes using CIBERSORT (https://cibersort.stanford.edu) [8, 13]. We applied CIBERSORT to examine the immune cell composition of GBM tissues in our study patients based on a validated leukocyte gene signature matrix containing 547 genes and 22 human immune cell subpopulations [14]. Gene expression profiles of GBM tissues from TCGA were entered into CIBERSORT for analysis, and the algorithm was run using the default signature matrix at 100 permutations. CD4 + T cells, CD8 + T cells, regulatory T cells (Tregs), B cells, and antigen-presenting cells (APCs) play a critical role in the GBM immune microenvironment [15, 16]. Therefore, we included eight representative immune cells for the study: CD8 + T cells, Tregs, naive CD4 + T cells, resting and activated memory CD4 + T cells, memory B cells, plasma B cells, and activated dendritic cells.

Pathway-based network analysis was performed based on the Search Tool for the Retrieval of Interacting Genes/Proteins (STRING) database version 11.0 (http://www.string-db.org/). To evaluate the correlation between *DKK3* and immune-related genes in the STRING database, we obtained whole gene sets associated with immune system processes from GSEA (https://www.gsea-msigdb.org/gsea/msigdb/cards/IMMUNE_SYSTEM_PROCESS). Overall, 332 genes associated with the immune system obtained from GSEA and DKK3 were entered into the STRING database. We used the confidence setting in STRING between network edges and activated all interaction sources, including text mining, experiments, databases, co-expression, neighborhood, gene fusion, and co-occurrence. The Markov cluster (MCL) algorithm was used to cluster the network (inflation parameter = 3.0), and the minimum required interaction score was set at 0.350, which implied that any interaction power between two proteins that was below the level of medium confidence was excluded from our analysis.

We also performed the pathway network analyses using Cytoscape (version 3.9.0) software (https://cytoscape.org/). To interpret the biological relevance of DKK3 and its relevant elements in GBM, we performed functional enrichment analysis to clarify functionally grouped gene ontology and pathway annotation networks using ClueGO (version 2.5.8) [17]. Using the GSEA, we obtained 335 genes related to DDK3 (arranged with the highest related score). We used an application (clueGO) that enabled functional ontology analysis in Cytoscape. We analyzed the biological function annotated pathways based on 335 genes related to DDK3 with DKK3, CD4, and CD8 genes.

### Data extraction from the GDSC and COSMIC databases

Drug screening was performed using datasets from the GDSC and COSMIC databases, which are large-scale cancer cell line and drug response databases containing data from 1,796 cancer cell lines and 565 compounds, respectively [18, 19]. The response of 49 GBM cell lines (cell lines with low DDK3 expression: U-87-MG, GI-1, YH-13, D-423MG, KNS-42, H4, GB-1, LN-18, DK-MG, D-263MG, LNZTA3WT4, D-336MG, SW1783, MOG-G-UVW, U-118-MG, CAS-1, D-566MG, LN-229, U251, D-502MG, SW1088, KS-1, T98G, no-11, SF539, GAMG, CCF-STTG1, M059J, A172, no-10, SNB75, GMS-10; DDK3 < 2 based on the z-score versus cell lines with high DDK3 expression: KALS-1, SF268, AM-38, D-392MG, SF126, Becker, SK-MG-1, D-247MG, MOG-G-CCM, LN-405, DBTRG-05MG, SF295, KINGS-1, KNS-81-FD, YKG-1, D-542MG, NMC-G1; DDK3 > 2 based on the z-score) to 316 drugs was measured and reported as the natural log half-maximal inhibitory concentration (LN IC50) value. A drug was defined as effective when the LN IC50 value was lower in GBM cell lines with high DKK3 expression than in those with low DKK3 expression.

### Machine learning algorithm for validation.

We applied machine learning (ML) algorithms to find the gene with the highest correlation with survival as follows: Genes Associated with Immune Cell Decrease: AKT1, ANKRD6, APC, CBY1, CSNK1A1, CXXC4, DACT1, DKK1, DKK3, DKK4, DVL1, FRAT1, FSTL1, GSK3A, GSK3B, LRP1, NIT2, PIN1, PSEN1, PTPRA, and SFRP. (Randomization: train set, 70%; validation set, 30%). A learning algorithm was independently applied to select and combine multiple covariates from gradient boosting machines (GBM) based on multivariate Gaussian models (Supplementary Data 2). In this step, ‘‘forward” search method, which initiates on a prototype set and selects a feature if and only if the addition of the feature could increase the performance of the prognostic model, is adopted to select optimal features sequentially. We did not consider hyperparameters tunning of the ML algorithm, because it designs to identify a high priority gene associated with survival rates.

### Statistical analysis

Student’s t test was used to evaluate differences between continuous variables. We performed Pearson’s correlation analysis to evaluate the relationships between the mRNA expression of 31 genes associated with the Wnt/β-catenin pathway and eight immune cell fractions. Pearson’s correlation coefficients and significance levels (*p-*values) were calculated between the Wnt/β-catenin-related gene sets and representative immune cells.

Overall survival (OS) and progression-free survival (PFS) were estimated by Kaplan–Meier analysis according to the DKK3 expression tertiles (tertile 1 = low expression; tertile 2 = moderate expression; tertile 3 = high expression). We then calculated hazard ratios (HRs) with 95% confidence intervals (CIs) using multivariate Cox regression analysis to determine whether DKK3 was independently associated with OS and PFS in GBM patients. Because the effect of adjuvant therapy (adjuvant chemotherapy and/or immunotherapy) is associated with the status of the immune microenvironment in GBM [20], we also estimated the OS and PFS according to the adjuvant therapy classified by DKK3 tertile (low expression, moderate expression and high expression).

A *p*-value < 0.05 was considered statistically significant. All statistical analyses were performed using R software version 4.1.1 and SPSS for Windows version 24.0 (IBM, Chicago, IL).

## Results

### Characteristics of the study patients

A total of 525 patients with GBM from TCGA database were included in the study. The mean patient age at diagnosis was 57.7 years, and 39.0% of patients were female. Radiation treatment was performed in 82.9% of patients, and 39.0% of patients received adjuvant therapy (adjuvant chemotherapy and/or immunotherapy). Further detailed information, including immune cell fractions in GBM tissues, is shown in Table 1.

**Table 1:** Clinical and immune cell characteristics in patients with GBM

Characteristics	Total
Number	525
Sex, female, n (%)	205 (39.0)
Age at diagnosis of GBM, mean ± SD, y	57.7 ± 14.6
Time duration between GBM diagnosis and death (days), mean ± SD	508.9 ± 539.4
Time duration between GBM diagnosis and disease progression (days), mean ± SD	307.0 ± 391.0
Karnofsky performance scale score, median (IQR)	80.0 (70.0–80.0)
Missing data, n (%)	133 (25.3)
Radiation treatment, n (%)	
Yes	435 (82.9)
No	70 (13.3)
Missing data	20 (3.8)
Adjuvant therapy, n (%)	
Yes	205 (39.0)
No	2 (0.4)
Missing data	318 (60.6)
IDH1 mutation status, n (%)	
Mutant	14 (2.7)
Wild-type	230 (43.8)
Missing data	281 (53.5)
History of prior glioma, n (%)	15 (2.9)
Immune cells (CIBERSORT fraction), mean ± SD	
CD8 + T cells	0.022 ± 0.039
Regulatory T cells	0.010 ± 0.022
Naive CD4 + T cells	0.006 ± 0.021
Resting CD4 + memory T cells	0.082 ± 0.067
Activated CD4 + memory T cells	0.003 ± 0.011
Memory B cells	0.031 ± 0.040
Plasma B cells	0.017 ± 0.039
Activated dendritic cells	0.016 ± 0.022

*GBM* glioblastoma multiforme, *SD* standard deviation, *IQR* interquartile range, *IDH* isocitrate dehydrogenase

### Correlation between gene expression and immune cell fraction

**Fig. 2:**
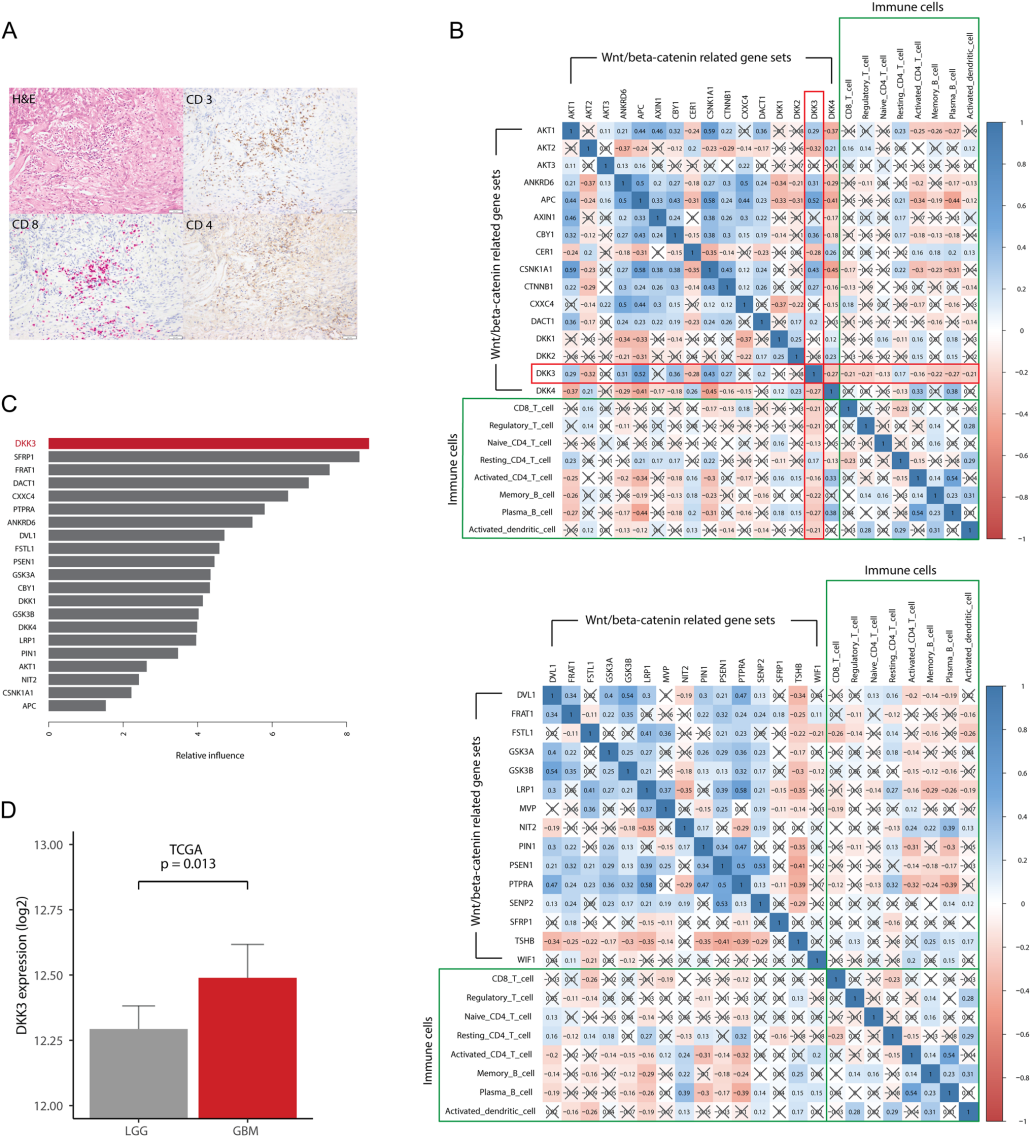
Microphotographs of GBM and correlation plots between Wnt/β-catenin-related gene sets and representative immune cells **A** Representative GBM microphotographs showing lymphocyte infiltrations stained with H&E (hematoxylin and eosin), CD3 + T cells (brown), CD8 + T cells (red) and CD4 + T cells (brown) from our hospital cohort; **B** Pearson correlation coefficients and significance levels were calculated between Wnt/β-catenin-related gene sets and representative immune cells. The color coordinated legend indicates the value and sign of Pearson’s correlation coefficient. The number in the box indicates Pearson’s correlation coefficient. Moreover, an x in the box indicates a p value ≥ 0.001. (Upper) Correlations between AKT1 and DKK4 among Wnt/β-catenin-related genes and representative immune cells; (Lower) correlations between DVL1 and WIF1 among Wnt/β-catenin-related genes and representative immune cells; **C** supervised machine-learning models for prognosis prediction using gradient boosting machine (GBM); **D** bar plots showing the difference in DKK3 expression between low grade glioma (LGG) and GBM

The mRNA expression levels of 31 genes associated with the Wnt/β-catenin pathway in GBM patients are shown in Supplementary Fig. S1. DKK3 showed the highest expression level among the DKK family members (DKK1-4), and the expression level of DKK3 was quite high among other Wnt/beta catenin-related genes. Microphotographs showing immune cell infiltration in GBM tissue is presented in Fig. 2A. We observed CD3 + T cells, CD8 + T cells, and CD4 + T cell infiltration in GBM tissues obtained from our hospital cohort. When we calculated correlations between the mRNA expression levels of 31 genes associated with Wnt/β-catenin signaling and 8 immune cell fractions in GBM patients, only DKK3 showed statistically significant correlations (*p* < 0.001) with all 8 immune cell fractions (an x in the box indicates a *p*-value ≥ 0.001) (Fig. 2B). DKK3 showed significant negative correlations with all immune cell fractions except for the resting CD4 + memory T cell fraction. Among the DKK family, there were significant positive correlations between DKK1, 2, and 4. However, DKK3 showed no significant correlations with DKK1 and DKK2 and a significant negative correlation with DKK4 (Fig. 2B). Supervised learning was conducted based on the survival period using 21 genes with decreased immune cells. DKK3 had the highest effect on survival (Fig. 2C). DKK3 expression was higher in GBM, a more severe cancer, than in LGG, a less severe cancer (Fig. 2D).

**Fig. 3:**
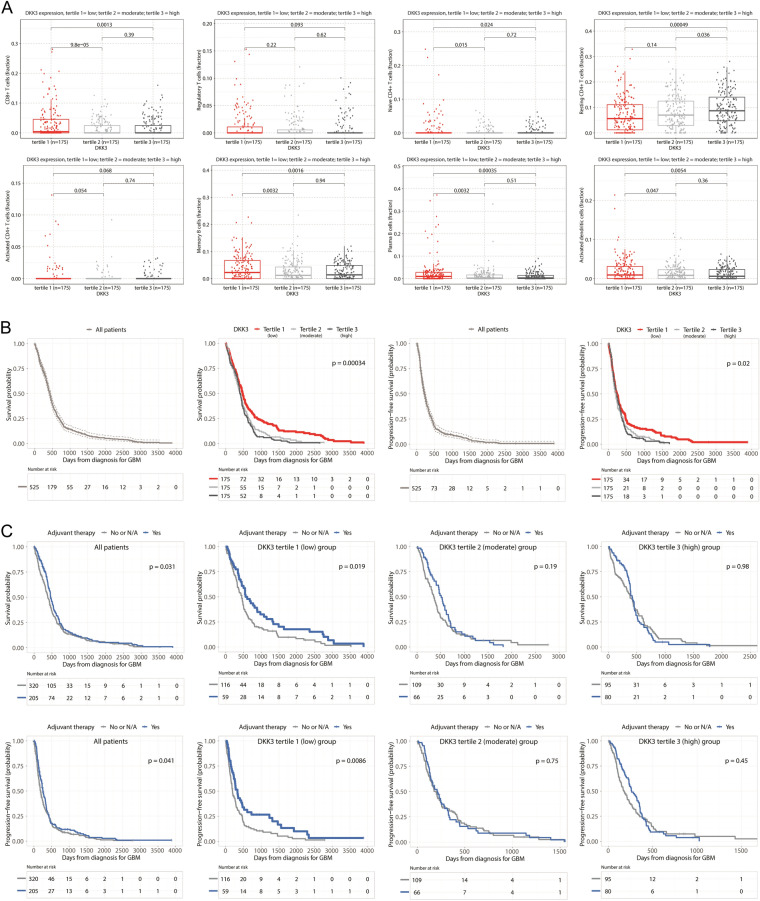
Comparison of CIBERSORT immune fractions and overall survival (OS) and progression-free survival (PFS) rates according to DKK3 expression level. Comparison of OS and PFS rates according to adjuvant therapy classified by DKK3 expression tertiles. **A** Boxplots with dot plots of CIBERSORT immune fractions of CD8 + T cells, Tregs, naive CD4 + T cells, resting and activated CD4 + memory T cells, memory B cells, plasma B cells, and activated dendritic cells based on DKK3 tertiles; **B** Kaplan–Meier curves showing the OS and PFS rates according to DKK3 tertiles; **C** Kaplan–Meier curves showing the OS and PFS rates according to adjuvant therapy based on DKK3 expression tertiles

When we divided patients into tertiles based on DKK3 expression, there were statistically significant differences in immune cell fractions for most immune cells between the DKK3 expression tertiles (Fig. 3A).

### Association between DKK3 expression and OS and PFS in patients with GBM

The GBM patients in the first DKK3 tertile showed significantly greater OS and PFS rates than those in the second and third tertiles (Fig. 3B). Multivariate Cox regression analysis showed that being in the first DKK3 expression tertile was an independent predictor of greater OS and PFS in GBM patients compared to patients in the highest tertile (HR, 0.73; 95% CI, 0.55–0.97; *p* = 0.031; HR, 0.75; 95% CI, 0.56–0.99; *p* = 0.044, respectively) (Table 2). When we also estimated the OS and PFS based on adjuvant therapy stratified by DKK3 expression tertile, we found that the received adjuvant therapy group showed significantly greater OS and PFS rates only among the first DKK3 tertile (Fig. 3C). We also present the OS and PFS based on the LRP1 and PTPRA tertiles in Supplementary Fig. S2 because they showed the second strongest correlations with immune cell fractions among gene sets related to the Wnt/β-catenin pathway after DKK3 (Fig. 2B).

**Table 2:** Overall survival and progression-free survival analyses according to clinical parameters and DKK3 expression in patients with GBM

Variable	Overall survival				Progression-free survival			
	Univariate analysis		Multivariate analysis		Univariate analysis		Multivariate analysis	
	HR (95% CI)	p	HR (95% CI)	p	HR (95% CI)	p	HR (95% CI)	p
Sex								
Male	Reference		Reference		Reference		Reference	
Female	0.86 (0.71–1.04)	0.122	0.85 (0.67–1.08)	0.191	0.85 (0.70–1.02)	0.085	0.89 (0.70–1.13)	0.338
Age at diagnosis of GBM (per 1 year increase)	1.03 (1.03–1.04)	** < 0.001**	1.03 (1.02–1.04)	** < 0.001**	1.02 (1.01–1.03)	** < 0.001**	1.01 (1.01–1.02)	**0.002**
Karnofsky performance scale score (per 10 score increase)	0.98 (0.97–0.98)	** < 0.001**	0.98 (0.97–0.99)	** < 0.001**	0.99 (0.98–1.00)	**0.014**	1.00 (0.99–1.00)	0.272
Radiation treatment (yes vs. no)	0.24 (0.19–0.32)	** < 0.001**	0.31 (0.21–0.44)	** < 0.001**	0.35 (0.26–0.47)	** < 0.001**	0.42 (0.27–0.66)	** < 0.001**
Adjuvant therapy (yes vs. no or N/A)	0.81 (0.67–0.98)	0.032	0.87 (0.69–1.10)	0.246	0.82 (0.68–0.99)	**0.042**	0.75 (0.60–0.94)	**0.014**
History of prior glioma (yes vs. no)	0.80 (0.45–1.42)	0.444	0.83 (0.41–1.69)	0.605	0.90 (0.52–1.57)	0.708	0.86 (0.44–1.69)	0.660
DKK3 tertiles								
Tertile 1	0.64 (0.50–0.80)	** < 0.001**	0.73 (0.55–0.97)	**0.031**	0.75 (0.60–0.95)	**0.016**	0.75 (0.56–0.99)	**0.044**
Tertile 2	0.91 (0.72–1.14)	0.389	0.89 (0.68–1.17)	0.411	1.01 (0.80–1.26)	0.958	1.00 (0.76–1.31)	0.986
Tertile 3	Reference		Reference		Reference		Reference	

*GBM* glioblastoma multiforme, *DKK* dickkopf-3 *HR* hazard ratio, *CI* confidence interval, *N/A* not available; *p* < 0.05 is shown in bold

Additionally, we calculated OS and PFS between the isocitrate dehydrogenase (IDH)1 mutant and the IDH1 wild-type groups among patients with IDH1 mutation information (*n* = 244). Furthermore, for patients with IDH1 wild-type, OS and PFS were compared according to the DKK3 tertile groups. We noted a statistically significant difference in OS rates between the DKK3 tertiles (*p* = 0.014) (Supplementary Fig. S3).

### Pathway-based network analysis

**Fig. 4:**
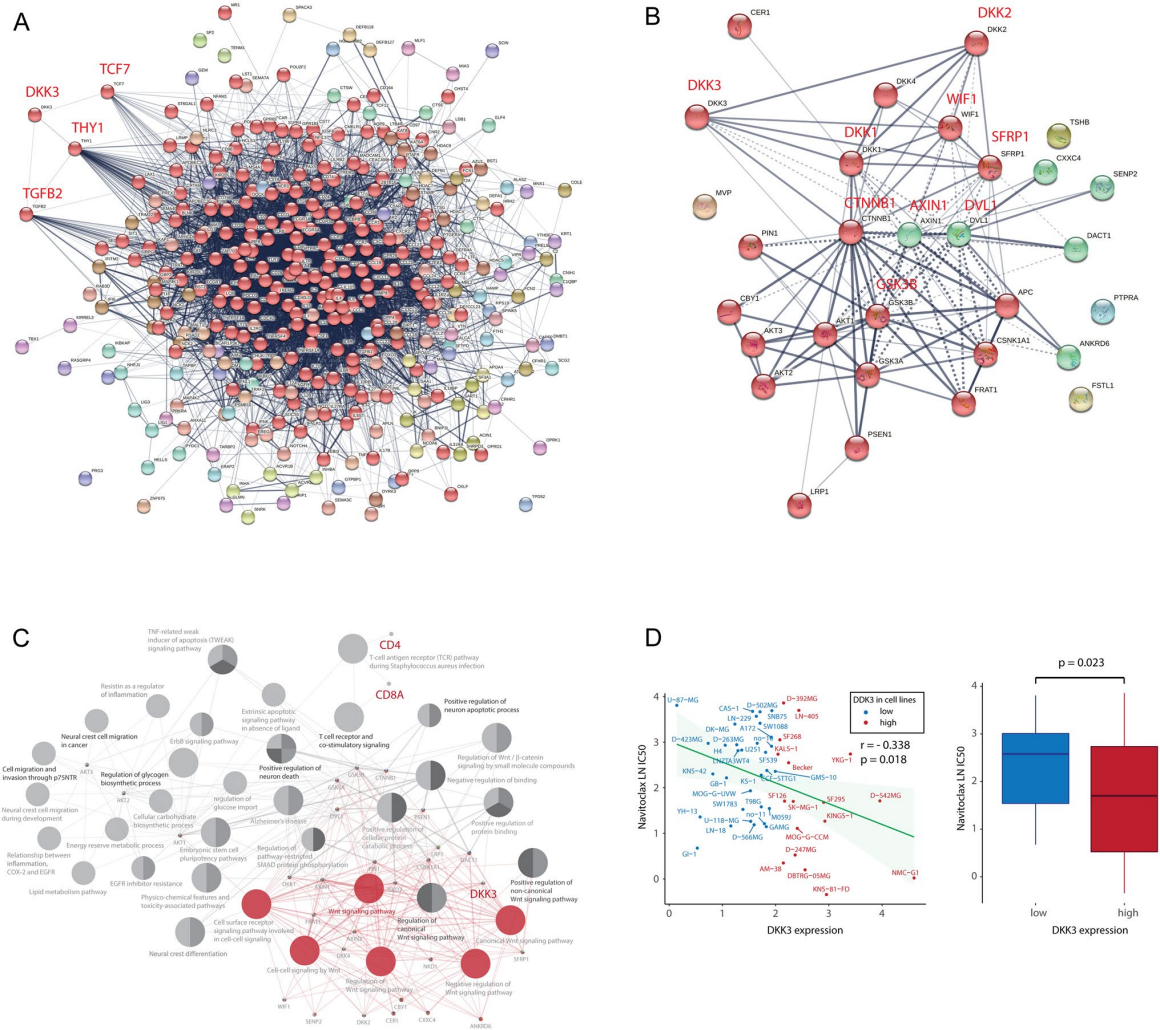
Bioinformatics analysis of DKK3 and Genomics of Drug Sensitivity in Cancer (GDSC) database analysis. **A** A protein–protein interaction network was constructed among the whole gene sets related to the immune system process; **B** a protein–protein interaction network was constructed among the gene sets associated with the Wnt/β-catenin pathway; **C** grouping of networks based on functionally enriched GO terms and pathways using Cytoscape software (version 3.9.0) and ClueGO application (version 2.5.8) (https://cytoscape.org/): DKK3 is indirectly linked to immune cell regulation (CD4 + and CD8 + T cells) as well as signal pathways related to migration, invasion and apoptosis; **D** Pearson’s correlation analysis showing the natural log of the half-maximal inhibitory concentration (LN IC50) values of navitoclax (ABT-263) in GBM cancer cell lines (red, low DKK3 expression; gray, high DKK3 expression). Boxplot showing the LN IC50 values of navitoclax (ABT-263) in GBM cancer cell lines based on low (red) and high (gray) DKK3 expression (*p* = 0.023)

Direct or indirect interactions were investigated between DKK3 protein and immune-related protein sets and protein sets associated with the Wnt/β-catenin pathway (Fig. 4A–C). We observed that the DKK3 protein interacted with three immune system-related proteins: TCF7 (transcription factor 7), THY1 (CD90), and TGFβ2 (transforming growth factor beta 2) (Fig. 4A). Among the interactions between the Wnt/β-catenin-related protein sets, DKK3 showed interactions with eight proteins: DKK1, DKK2, WIF1 (WNT inhibitory factor 1), SFRP1 (secreted frizzled-related protein 1), DVL1 (dishevelled segment polarity protein 1), AXIN1 (axis inhibition protein 1), CTNNB1 (catenin, beta-1), and GSK3B (glycogen synthase kinase 3 beta) (Fig. 4B). In functionally grouped network analysis, we found that DKK3 was indirectly linked to immune cell regulation (CD4 + and CD8 + T cells) as well as signal pathways related to migration, invasion, and apoptosis (Fig. 4C).

We also analyzed OS and PFS in the tertiles based on those 11 proteins in the GBM patients in the study (Supplementary Fig. S4 and S5). Only the PFS of DKK1 was significant (*p* = 0.046), and none of the others was significant (Supplementary Fig. S5).

Using TCGA data, we performed GSEA to identify the CD8 + T cell-related gene sets associated with high DKK3 expression. We found that high DKK3 expression was associated with 32 downregulated and 30 upregulated gene sets linked to CD8 + T cells (Supplementary Fig. S6).

### Drug screening in GBM cell lines with high DKK3 expression

Based on the GDSC data, we analyzed drug sensitivity patterns to 565 drugs in 49 GBM cell lines with high DKK3 expression. We considered drugs with a high negative correlation between DKK3 expression and the LN IC50 value to be effective DKK3-targeting drugs. Navitoclax (ABT-263) reduced the growth of cancer cell lines with high DKK3 expression (*r* = −0.338, *p* = 0.018 [Pearson’s correlation] and 0.023 [Student’s t test]) (Fig. 4D).

## Discussion

Cancer stemness is associated with a suppressed immune response and higher intratumoral heterogeneity, leading to treatment resistance and dramatically worse outcomes for the majority of cancer patients [3]. Wnt/β-catenin is critical for stemness activation and resistance to temozolomide (TMZ) chemotherapy in GBM [6, 7]. Glioblastoma stem cells downregulate the expression of major histocompatibility complex (MHC) molecules to escape tumor antigen-cognate T lymphocytes, thus inducing an immunosuppressive tumor microenvironment [21, 22]. Therefore, as described in the Introduction, we hypothesized that high expression of Wnt/β-catenin-associated genes, especially those related to immunosuppression, may be associated with mortality and poor prognosis in GBM patients.

Contrary to our findings, several previous studies have reported that DKK3 showed antitumor effects and reduced chemoresistance in GBM [23, 24]. However, according to our results, among Wnt/β-catenin-associated genes, DKK3 was most significantly associated with immune suppression in GBM patients based on the public TCGA database, which is an open source and freely available for downloads. However, although DKK3 expression is significantly related to the overall immune suppression condition in GBM tissue, we do not know why DKK3 is negatively correlated with regulatory T cells and positively associated with resting CD4 + memory T cells. Regulatory T cells are known to suppress T cell activation and immune responses [25]. However, previous studies have reported that DKK3 is associated with immunosuppression [26–28]. Therefore, we believe that there may be complex immune reactions associated with DKK3 that have not yet been elucidated in GBM. In addition, interestingly, our study showed that higher DKK3 expression in GBM was significantly associated with a higher risk of mortality and disease progression than lower DKK3 expression. In addition, only among the lower DKK3 expression group did patients receiving adjuvant chemotherapy or immunotherapy show a higher survival rate and decreased disease progression compared to patients with no or unknown adjuvant chemotherapy or immunotherapy treatment history. Therefore, we believe that *DKK3* may play an important role in GBM progression and mortality and predict clinical outcomes in GBM patients.

Whether DKK3 promotes or inhibits the Wnt/β-catenin signaling pathway remains controversial [29]. Based on our findings, positive correlations were shown between DKK1, 2, and 4 in GBM patients. However, despite being in the same DKK family, DKK3 showed no such correlations with DKK1, 2, and 4. Therefore, we think that DKK3 may play different roles in GBM than DKK1, 2, and 4. Previous studies have reported that DKK1 inhibits Wnt/β-catenin signaling, whereas DKK3 promotes Wnt/β-catenin signaling [30, 31]. In studies using TCGA data such as ours, DKK3 was also associated with poor prognosis in head and neck squamous cell carcinoma, pancreatic cancer, and renal cancer [32]. In addition, although DKK3 is known to have a tumor suppressive effect, DKK3 was well expressed in GBM in our study and was recently reported to have an oncogenic effect [32, 33]. Based on the protein–protein interactions in protein sets associated with the Wnt/β-catenin signaling pathway, DKK3 protein showed interactions with DKK1, DKK2, WIF1, SFRP1, DVL1, AXIN1, CTNNB1, and GSK3B. However, in the study, there were no significant differences in survival rate and disease progression according to the expression levels of those genes except for the disease progression related to DKK1. According to our study, among Wnt/β-catenin-related genes, *DKK3* was uniquely associated with both survival and disease progression in GBM patients.

It is well known that DKK3 suppresses CD8 + and CD4 + T cell-mediated responses [26–28]. We observed that DKK3 expression is also associated with downregulation of immune responses in GBM tissue. To the best of our knowledge, this study is the first to show that *DKK3* is possibly associated with an immunosuppressive GBM microenvironment. According to the protein–protein interactions in protein sets related to the immune system, DKK3 protein was associated with the TCF7, THY1, and TGFβ2 proteins. TCF7 promotes cell proliferation by increasing c-Myc expression in GBM [34]. c-Myc plays a critical role in regulating the proliferation and survival of glioma cancer stem cells [35]. THY1 (CD90) expression is associated with glioma cell proliferation and invasiveness [36]. In addition, THY1 (CD90) expression is a marker of cancer stem cells in high-grade gliomas [37]. The TGF-β pathway has been reported to be associated with cell proliferation, invasion, angiogenesis, immunosuppression, and maintenance of stemness of glioma cancer stem cells [38]. TCF7, THY1, and TGFβ2 are all associated with glioma stemness, leading to immunosuppression. According to our study, DKK3 expression also showed immune suppression in GBM. Therefore, we thought that DKK3, TCF7, THY1, and TGF β2 would all have similar immunosuppression-related actions in GBM. However, there were no significant differences in mortality or disease progression between the TCF7, THY1, and TGFβ2 expression GBM patient tertiles. Therefore, we suspect that there may be unknown important pathways linked to DKK3 that significantly affect mortality and disease progression in patients with GBM.

**Fig. 5:**
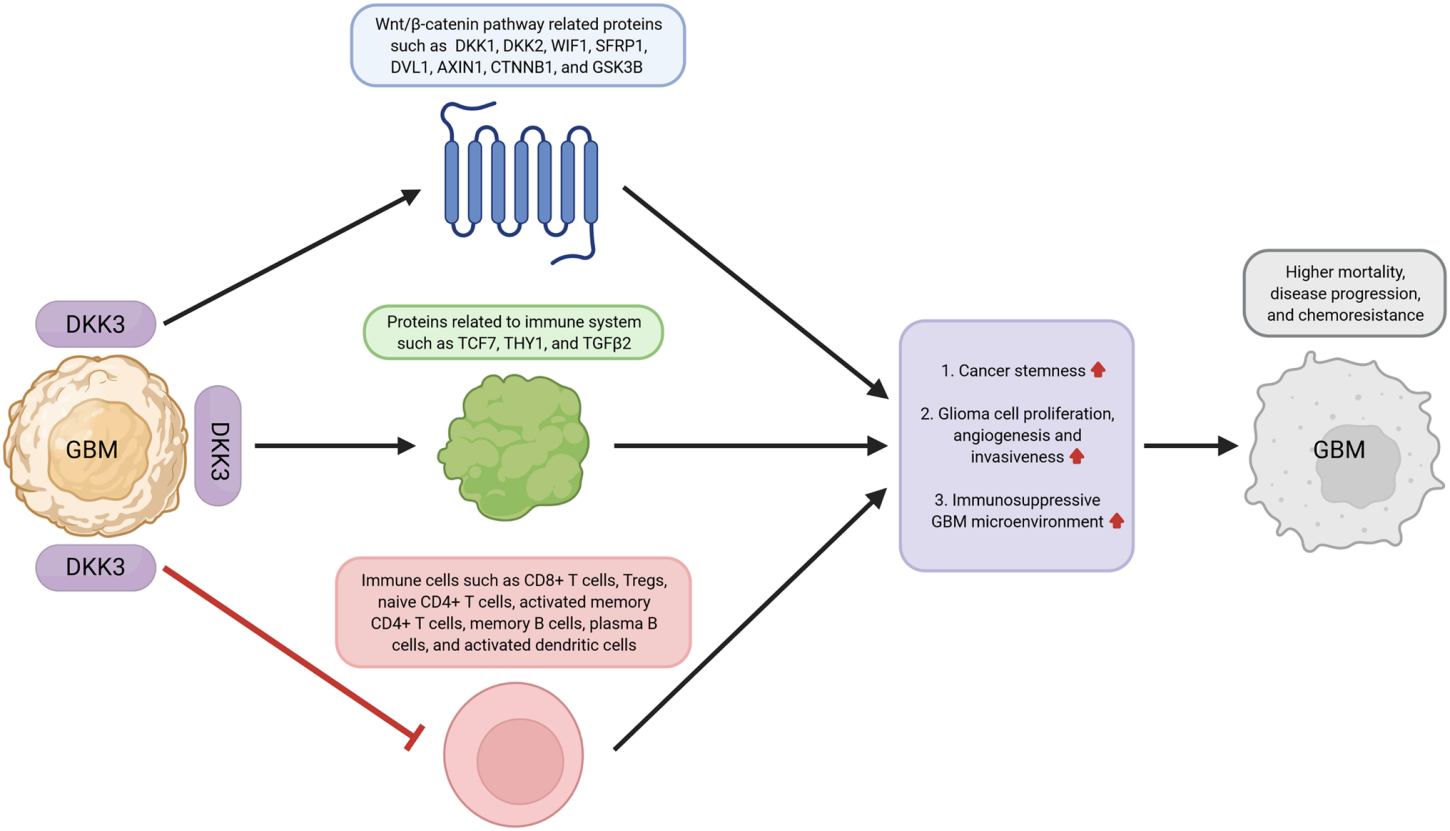
A schematic diagram presenting the proposed mechanisms underlying the effect of DKK3 on GBM. DKK3 interacts with proteins related to the Wnt/β-catenin pathway and immune system. Immune cells in GBM may induce cancer stemness, glioma cell proliferation, and an immunosuppressive GBM microenvironment, resulting in increased mortality, disease progression, and chemoresistance in patients with GBM. GBM, glioblastoma

We also observed that adjuvant therapy was more effective when the DKK3 expression level was low in patients with GBM. The chemotherapeutic drug temozolomide for GBM is known to influence the immune system [39]. High-dose temozolomide may induce lymphopenia and T and B cell dysfunction [39]. The immunosuppressive glioma microenvironment also limits the effectiveness of immunotherapy [21]. Maintenance of antitumor immunity is important to enhance survival in GBM patients because the immune system recognizes cancer antigens and eradicates pathogens to maintain homeostasis [40]. Therefore, when antitumor immunity is better in the GBM microenvironment, adjuvant chemo- or immunotherapy will be more effective for improving survival and suppressing disease progression in patients with GBM. To summarize our findings, DKK3 interacts with proteins related to the Wnt/β-catenin pathway, such as DKK1, DKK2, WIF1, SFRP1, DVL1, AXIN1, CTNNB1, and GSK3B, and could promote cancer stemness in GBM. Cancer stemness is known to promote tumor growth, tumor cell heterogeneity, and the induction of immunosuppression [4]. In addition, DKK3 interacts with immune system-related proteins, including TCF7, THY1, and TGFβ2, and induces proliferation, angiogenesis, invasiveness, and immunosuppression of glioma cells. Furthermore, high expression of DKK3 may induce an immunosuppressive GBM microenvironment. We believe that all these underlying mechanisms work collectively and potentially lead to increased mortality, disease progression, and chemoresistance in patients with GBM (Fig. 5).

Through the GDSC database containing information on drug screening of cancer cell lines, we were able to investigate drug sensitivity in GBM cell lines according to DKK3 expression. Navitoclax reduced the growth of GBM cancer cell lines exhibiting high DKK3 expression. Navitoclax (ABT-263) is an orally available agent that is an inhibitor of bcl-2 and bcl-XL [41]. A previous study reported that combined treatment with a phosphatidylinositol 3-kinase (PI3K) inhibitor and navitoclax (ABT-263) markedly reduced cell viability and induced apoptotic cell death in GBM cell lines [42]. Wnt/β-catenin signaling is known to induce expression of the antiapoptotic/survival gene bcl-2 [43]. In addition, bcl-XL knockdown selectively reduced the viability of senescent-like GBM cells after radiation or temozolomide therapy [44]. Dual inhibition of Bcl-2/Bcl-xL and XPO1 potently induced GBM cell death with features of apoptosis in a highly synergistic manner [45]. Therefore, we think that navitoclax (ABT-263) may contribute to an improved treatment strategy for GBM with resistance to chemotherapy and high DKK3 expression.

This study has several limitations. First, we obtained all patient clinicopathological data from TCGA database, and there will be a certain bias due to the potential influence of confounding factors in TCGA database. Because all study samples were collected from a retrospective database, further prospective studies are needed to validate the results. However, since we used TCGA public data, we have the strength to enable researchers to transparently check and verify our results. Second, the fraction of immune cells was evaluated only with in silico flow cytometry-based analysis of immune-related genes, and thus, there may be a difference in the real number of immune cells. Third, an experimental analysis of the association between DKK3 expression and immune cells among GBM cells was not performed, and further in vitro and/or in vivo studies are necessary. Fourth, missing data, especially in adjuvant therapy, in TCGA database may influence the statistical analysis results in the study. Fifth, given the limited information on IDH1 mutations in GBM in the TCGA database, we could not comprehensively determine whether prognosis differs according to DKK3 expression between the IDH1 mutant and the IDH1 wild-type groups. We plan to evaluate differences in OS and PFS rates according to DKK3 expression levels between the IDH1 mutant and IDH1 wild-type groups using our hospital data in future investigations. Last, the relationship between DKK3 expression by GBM molecular subtype and survival and disease progression was not analyzed.

In conclusion, despite these limitations, using a large-scale open database, our study revealed that high DKK3 expression in GBM is associated with the immunosuppressive GBM microenvironment. High DKK3 expression in GBM tissue was also related to higher mortality, disease progression, and chemoresistance than low DKK3 expression in patients with GBM. In addition, we identified that navitoclax (ABT-263) affected GBM cancer cell lines with high DKK3 expression. We believe that these findings may suggest the possibility of DKK3 as a therapeutic target in GBM. We also believe that our findings will contribute to designing future clinical and experimental studies for patients with GBM.

## Supplementary Information

Below is the link to the electronic supplementary material.

Supplementary file1 (PDF 2436 KB)

## Data Availability

The authors declare that the data supporting the findings of this study are available from the corresponding author upon request.

## Abbreviations

APC: Antigen-presenting cell
CI: Confidence interval
CNS: Central nervous system
COSMIC: Catalog of somatic mutations in cancer
DKK3: Dickkopf-3
FDR: False discovery rate
GBM: Glioblastoma multiforme
GDSC: Genomics of drug sensitivity in cancer
GSEA: Gene set enrichment analysis
HR: Hazard ratio
MCL: Markov cluster
MHC: Major histocompatibility complex
OS: Overall survival
PFS: Progression-free survival
PI3K: Phosphatidylinositol 3-kinase
STRING: Search tool for the retrieval of interacting genes/proteins
TCGA: The cancer genome atlas
TMZ: Temozolomide
