# Supplementary material for: High DKK3 expression related to immunosuppression was associated with poor prognosis in glioblastoma: machine learning approach
Source: Cancer Immunol Immunother. 2022 May 23;71(12):3013–27. doi: 10.1007/s00262-022-03222-4 (PMC9588473; doi:10.1007/s00262-022-03222-4)
Supplement: Supplementary file 1 — Supplementary file1 (PDF 2436 KB) [file 262_2022_3222_MOESM1_ESM.pdf]

Supplementary Materials

High DKK3 expression related to immunosuppression was associated with poor prognosis in glioblastoma: machine learning approach

Authors: Myung-Hoon Han, Kyueng-Whan Min, Yung-Kyun Noh, Jae Min Kim, Jin Hwan Cheong, Je Il Ryu, Yu Deok Won, Seong-Ho Koh, Jae Kyung Myung, Ji Young Park, Mi Jung Kwon

Corresponding Author

Kyueng-Whan Min, M.D., Ph.D.

Department of Pathology, Hanyang University Guri Hospital, Hanyang University College of Medicine, Kyoungchun-ro 153, Guri-si, Gyeonggi-do 11923, Republic of Korea

Tel: +82-31-560-2496; Fax: +82-31-560-2339

E-mail: kyueng@gmail.com

ORCID ID: 0000-0002-4757-9211

Yung-Kyun Noh, M.D., Ph.D.

Department of Computer Science, Hanyang University, 605 IT/BT, 222 Wangsimni-ro, Seongdong-gu, Seoul 04763, Republic of Korea

Tel: +82-2-2220-1409; Fax: +82-2-2220-0397

E-mail: nohyung@hanyang.ac.kr

ORCID ID: 0000-0002-6372-9267

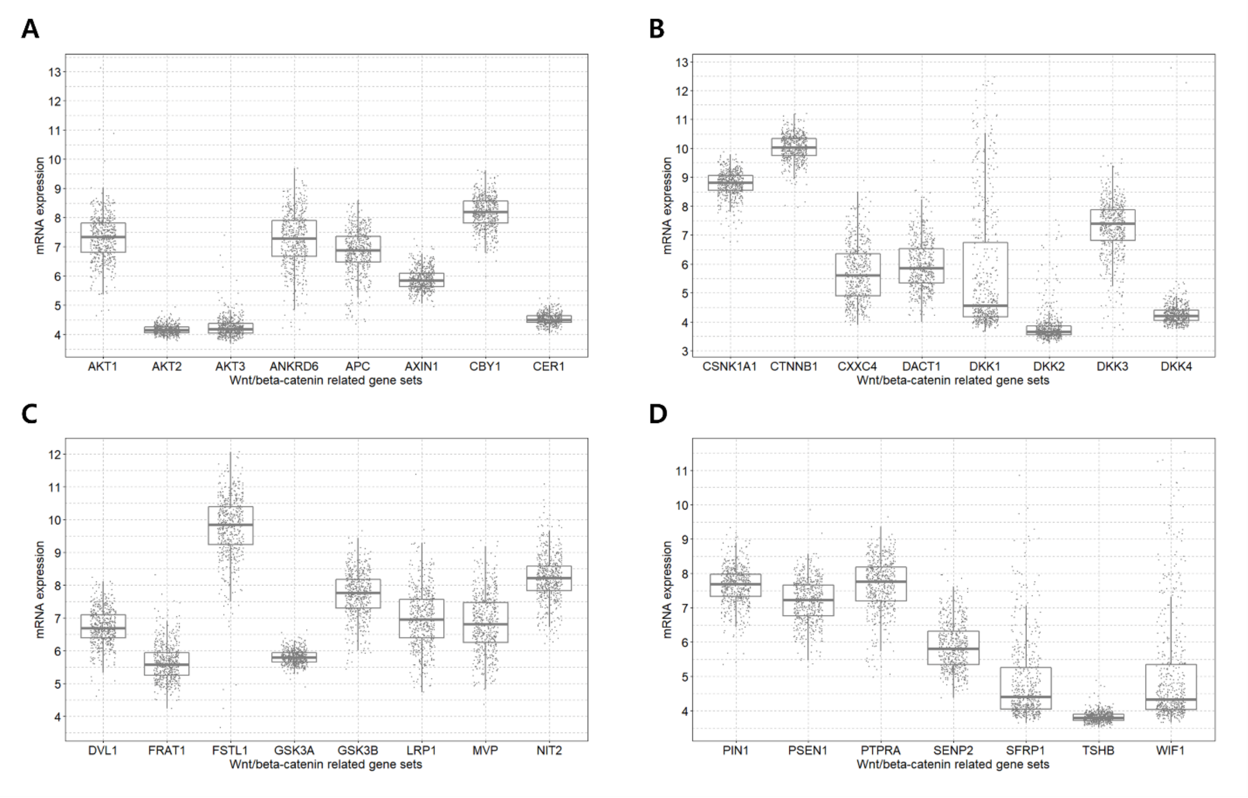

Supplementary Fig. S1. Box plots showing gene mRNA expression based on Wnt/β-catenin-related genes. (A) Gene mRNA expression of AKT1 to CER1 among Wnt/β-catenin-related genes; (B) gene mRNA expression of CSNK1A1 to DKK4 among Wnt/β-catenin-related genes; (C) gene mRNA expression of DVL1 to NIT2 among Wnt/β-catenin-related genes; (D) gene mRNA expression of PIN1 to WIF1 among Wnt/β-catenin-related genes.

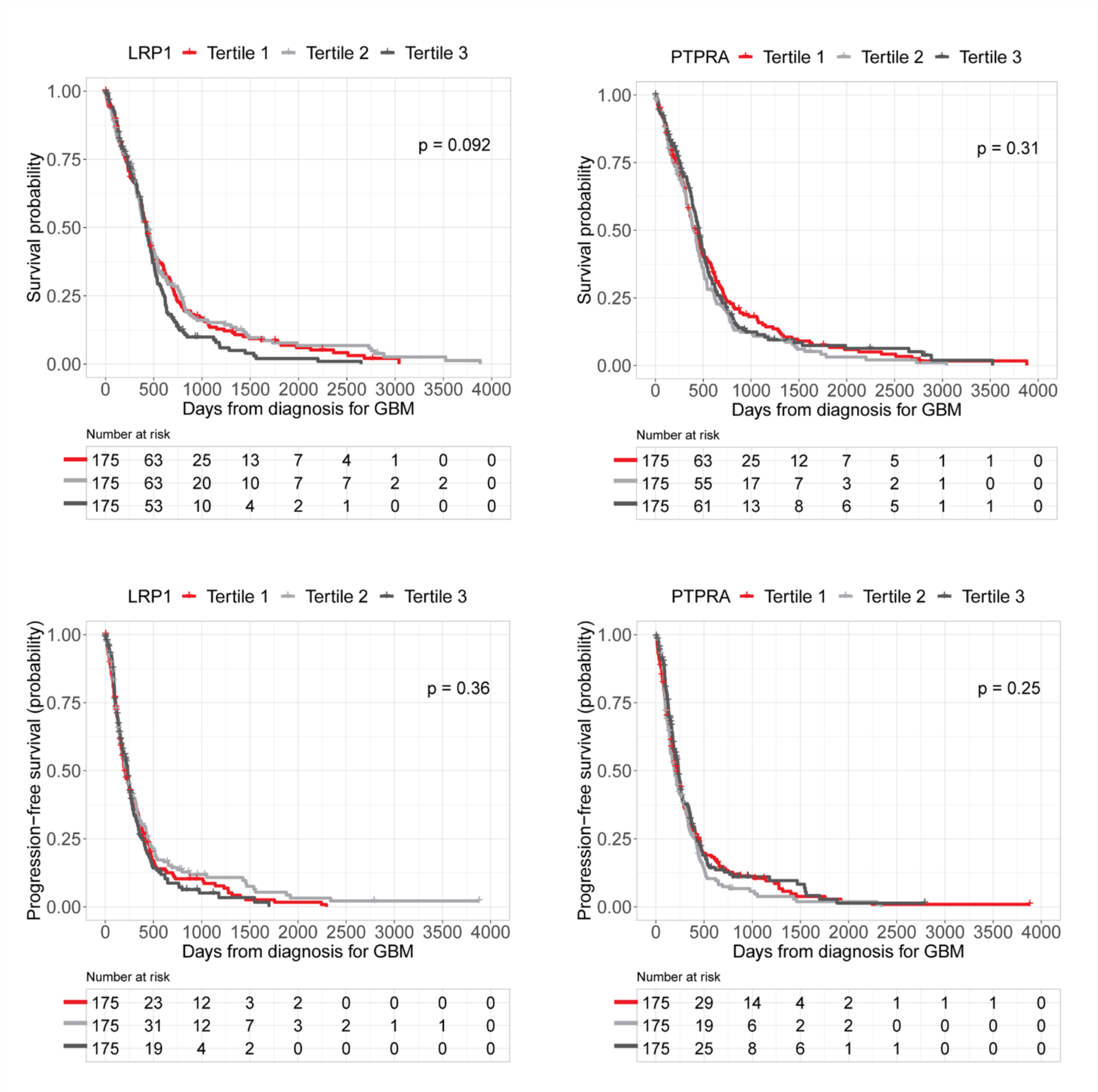

Supplementary Fig. S2. Kaplan–Meier curves showing overall survival (OS) and progression-free survival (PFS) rates according to LRP1 and PTPRA expression levels.

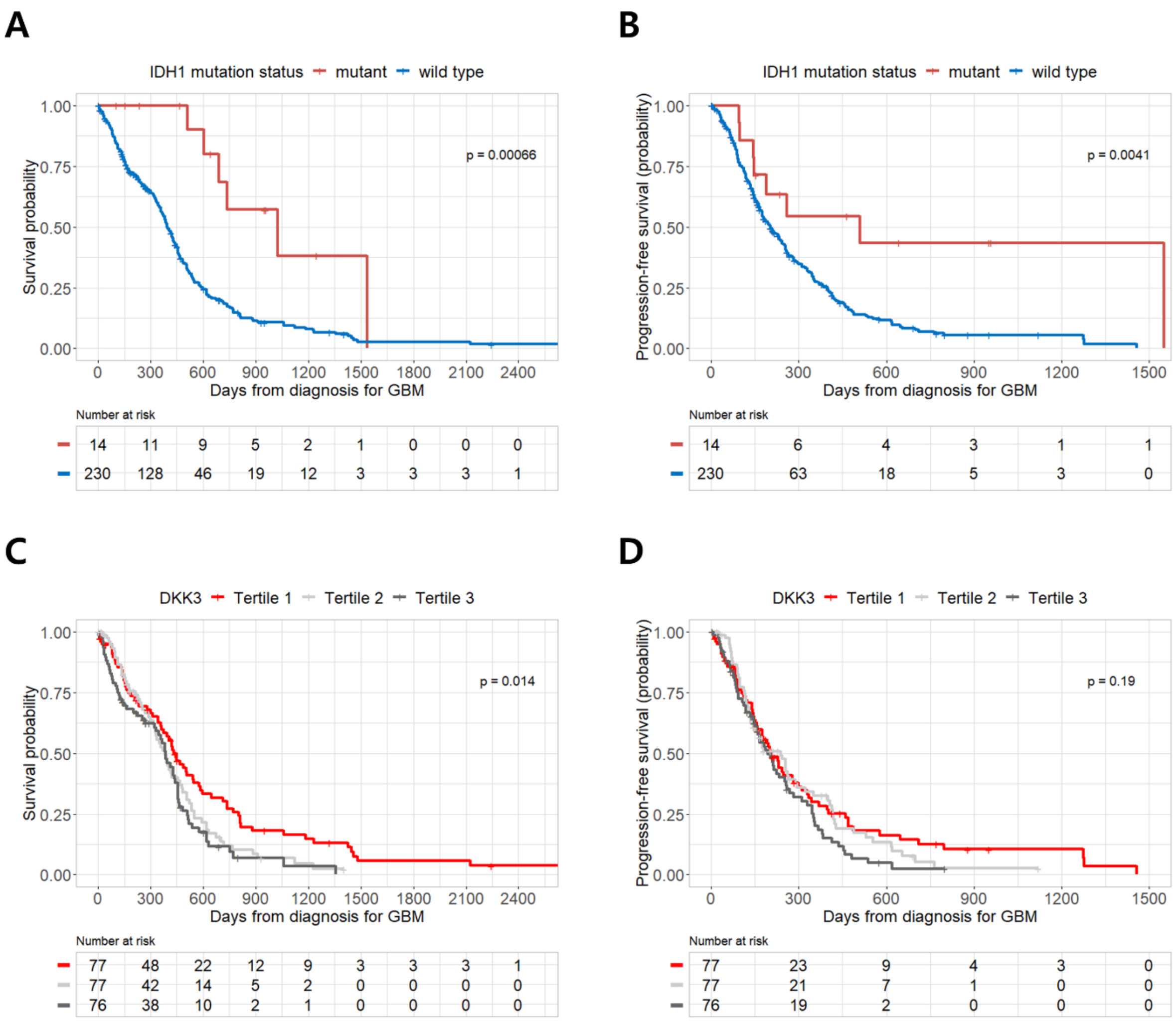

Supplementary Fig. S3. Kaplan–Meier curves showing overall survival (OS) and progression-free survival (PFS) rates. (A) OS rates according to IDH1 mutation status; (B) PFS rates according to IDH1 mutation status; (C) OS rates according to DKK3 tertiles in patients with IDH1 wild-type; (D) PFS rates according to DKK3 tertiles in patients with IDH1 wild-type. IDH1, isocitrate dehydrogenase 1.

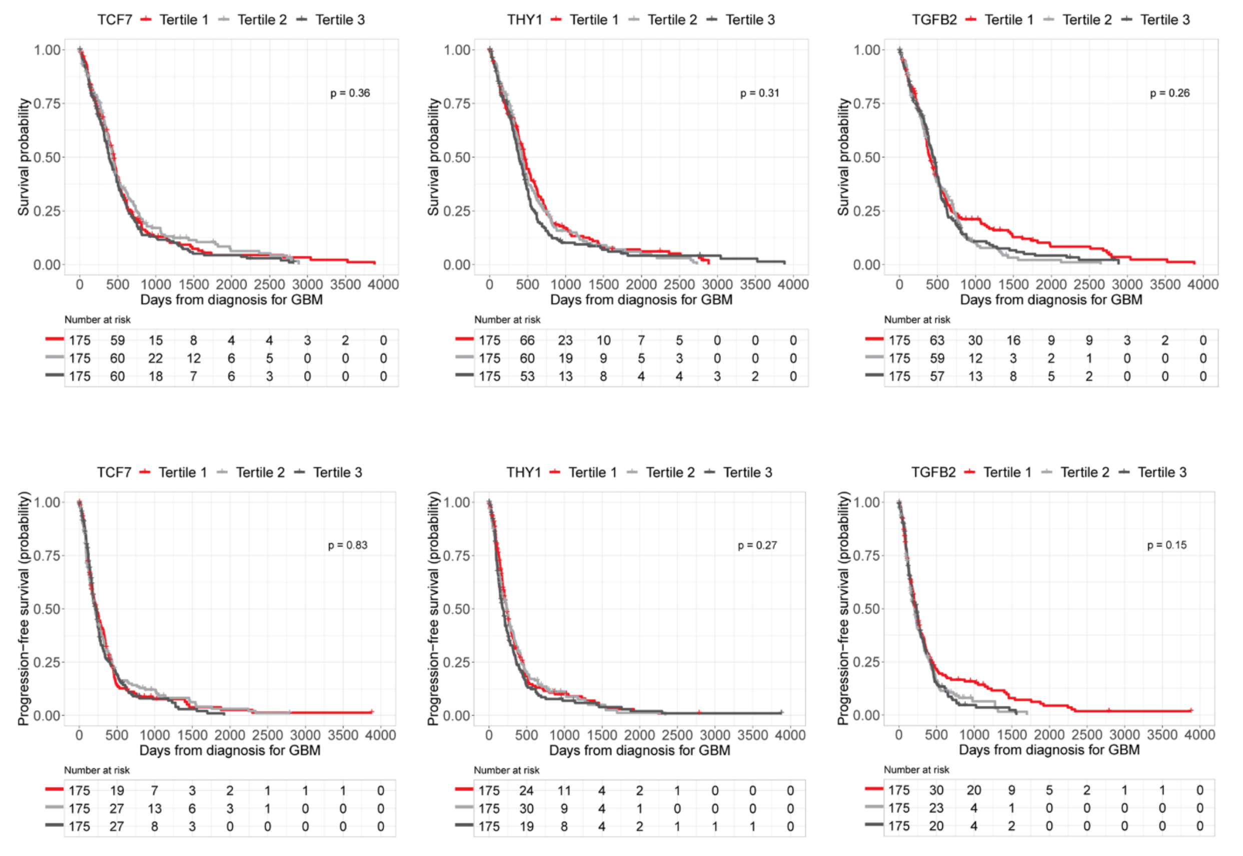

Supplementary Fig. S4. Kaplan–Meier curves showing overall survival (OS) and progression-free survival (PFS) rates according to TCF7, THY1 (CD90), and TGFβ2 expression levels.

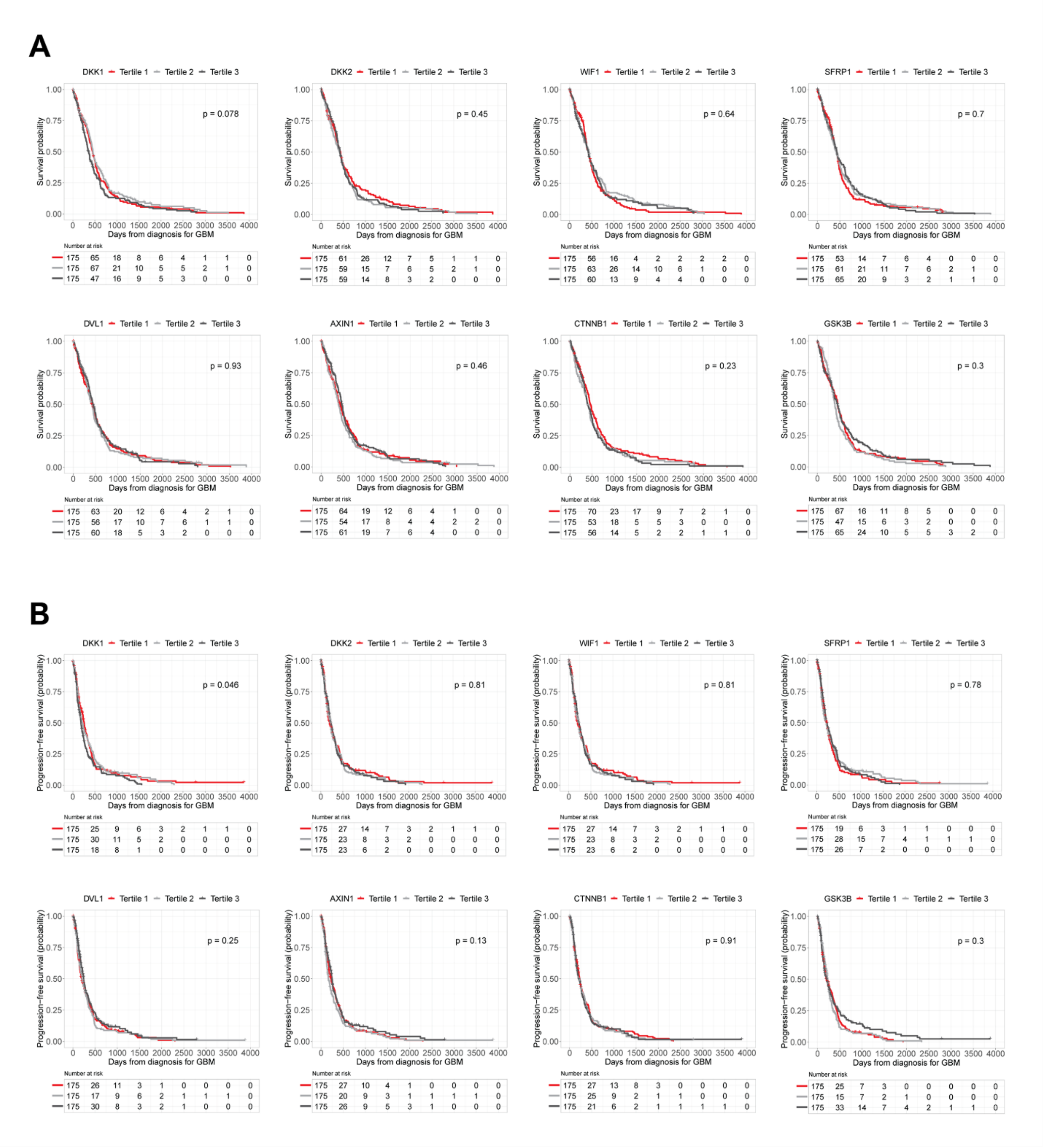

Supplementary Fig. S5. Kaplan–Meier curves showing overall survival (OS) and progression-free survival (PFS) rates according to DKK1, DKK2, SFRP1, DVL1, AXIN1, CTNNB1, and GSK3B expression levels. (A) OS rates based on the DKK1, DKK2, SFRP1, DVL1, AXIN1,

CTNNB1, and GSK3B expression tertiles; (B) PFS rates based on the DKK1, DKK2, SFRP1, DVL1, AXIN1, CTNNB1, and GSK3B expression tertiles.

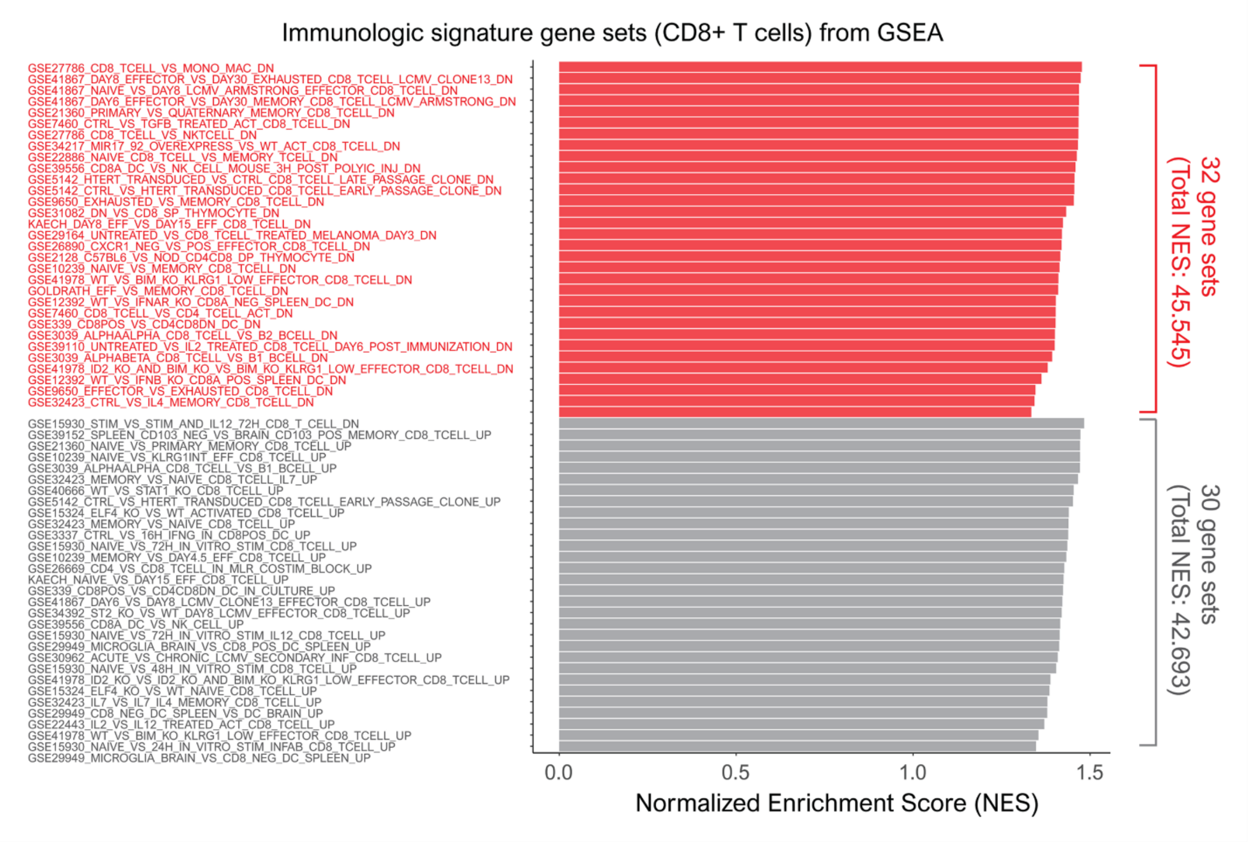

Supplementary Fig. S6. Gene set enrichment analysis of CD8+ T cell-related immunologic gene sets associated with high DKK3 expression.
